# Targeting PTEN Regulation by Post Translational Modifications

**DOI:** 10.3390/cancers14225613

**Published:** 2022-11-15

**Authors:** Ana González-García, Antonio Garrido, Ana C. Carrera

**Affiliations:** Department of Immunology and Oncology, Centro Nacional de Biotecnología, Consejo Superior de Investigaciones Científicas (CSIC), Universidad Autónoma de Madrid, Cantoblanco, E-28049 Madrid, Spain

**Keywords:** PTEN, post-translational modification, ubiquitination, sumoylation, phosphorylation

## Abstract

**Simple Summary:**

Cancer cells accumulate genetic alterations that improve their proliferation, survival, and migration capabilities. One of the most frequently altered signaling nodes in human cancer is the PI3-kinase /PTEN pathway. Most therapeutic efforts thus far have focused on the inhibition of PI3-kinase; however, a high proportion of tumors present an impaired activation of PTEN. While in some cases this is due to PTEN loss or inactivating mutations, PTEN activity can also be modulated by post-transcriptional modifications (PTMs). In this review, we discuss how these different modifications affect PTEN activity, and propose strategies to modulate these PTMs as an alternative approach for therapeutic treatment of PTEN-dependent tumors possessing at least one wild-type allele.

**Abstract:**

Phosphatidylinositol-3,4,5-triphosphate (PIP_3_) is a lipidic second messenger present at very low concentrations in resting normal cells. PIP_3_ levels, though, increase quickly and transiently after growth factor addition, upon activation of phosphatidylinositol 3-kinase (PI3-kinase). PIP_3_ is required for the activation of intracellular signaling pathways that induce cell proliferation, cell migration, and survival. Given the critical role of this second messenger for cellular responses, PIP_3_ levels must be tightly regulated. The lipid phosphatase PTEN (phosphatase and tensin-homolog in chromosome 10) is the phosphatase responsible for PIP_3_ dephosphorylation to PIP_2_. PTEN tumor suppressor is frequently inactivated in endometrium and prostate carcinomas, and also in glioblastoma, illustrating the contribution of elevated PIP_3_ levels for cancer development. PTEN biological activity can be modulated by heterozygous gene loss, gene mutation, and epigenetic or transcriptional alterations. In addition, PTEN can also be regulated by post-translational modifications. Acetylation, oxidation, phosphorylation, sumoylation, and ubiquitination can alter PTEN stability, cellular localization, or activity, highlighting the complexity of PTEN regulation. While current strategies to treat tumors exhibiting a deregulated PI3-kinase/PTEN axis have focused on PI3-kinase inhibition, a better understanding of PTEN post-translational modifications could provide new therapeutic strategies to restore PTEN action in PIP_3_-dependent tumors.

## 1. Introduction

**Figure 1:**
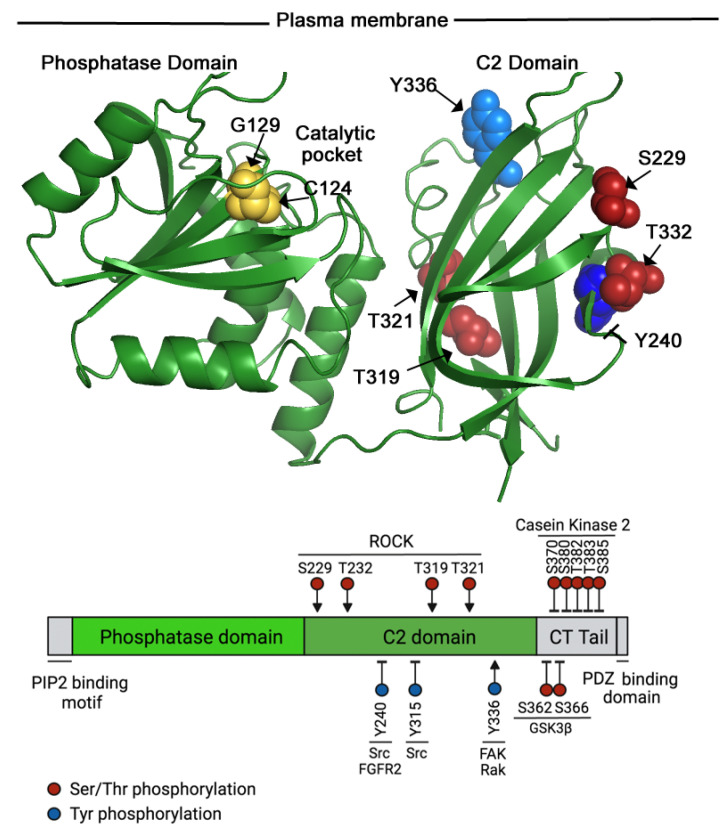
Phosphorylation at C2 domain residues can activate or inhibit PTEN; PTEN phosphorylation at the CT-tail is always inhibitory. The figure shows PTEN’s crystal structure (NCBI, https://www.ncbi.nlm.nih.gov/Structure/pdb/1D5R) [3], facing the plasma membrane (**top**), as well as a diagram showing PTEN’s domains (**bottom**). Two residues of the catalytic pocket are shown on the structure (in yellow). The different domains are indicated, as well as the Tyr (blue) or Ser/Thr (red) residues susceptible to phosphorylation (in the diagram and the structure). Phosphorylation might activate (residues indicated with an arrow) or inhibit (arrows ended with a line) PTEN activity. The kinases mediating PTEN phosphorylation are shown in the diagram. The N-terminal PIP_2_-binding motif, as well as the CT-tail and the PDZ-binding domains, are only shown in the diagram (in grey), as these are not resolved in the structure. Likewise, CT-tail inhibitory phosphorylation sites are shown only in the diagram.

PTEN (whose gene is located on chromosome 10q23) was first identified in 1997 as a phosphatase that is lost or mutated in several cancers [1,2]. PTEN is a 403-amino-acid polypeptide containing an N-terminal phosphatase domain (185 residues) and a C2 domain that mediates binding to the plasma membrane (see Figure 1). The N-terminal phosphatase domain adopts an α-helix-enriched structure, whereas the C2 domain exhibits the typical β-sheet structure of C2-domains [3]. At its very C-terminus, PTEN has a disordered region named the C-terminal (CT)-tail, whose structure has not been resolved. Recently, a 173-amino-acid-longer PTEN-long form generated by alternative initiation of translation has been reported; this 173aa N-terminal-tail permits PTEN-long to be secreted without impairing its phosphatase activity [4,5].

The high number of loss-of-function mutations in PTEN in cancer already suggests its tumor suppressor nature [1,2]. Several pieces of evidence have indicated that the tumor suppressor function of PTEN is related to its capacity to downregulate PIP_3_ levels [6,7]. Moreover, the similar phenotype of mice expressing an active allele of PI3-kinase on T cells [8] compared with that of mice showing PTEN loss in T cells [9] confirms a PIP_3_-related PTEN tumor suppressor function in vivo. Despite its lipid phosphatase activity being the main component of PTEN’s tumor suppressor function, PTEN also exhibits protein phosphatase activity towards a few substrates [10,11]. Whereas cytoplasmic PTEN is primarily involved in regulating PIP_3_ levels at the membrane, nuclear PTEN exhibits phosphatase-independent tumor suppressive functions, e.g., regulation of chromosome stability, DNA repair, and apoptosis [12,13].

Focusing on PIP_3_ regulation, in mammalian genomes, up to eleven genes encode for different isoforms of the catalytic and regulatory subunits of PI3-kinase [14], whereas mainly one gene product, PTEN, reduces PIP_3_ levels. As a consequence, cells must have different molecular mechanisms available to modulate the activity of PTEN. 

PTEN is frequently altered at the genetic level, and either somatic alterations (e.g., in tumor cells) or germline mutations have been described. The latter are responsible for a spectrum of clinical syndromes collectively known as PTEN Hamartoma Tumor Syndrome (PHTS). PHTS patients develop multiple benign tumors and have an increased lifetime risk of developing cancer, especially breast, thyroid, kidney, and endometrial cancers [15]. PTEN somatic alterations include gene mutation and gene loss; loss of heterozygosity is observed at high frequencies in cancer cells, while homozygous deletion is also detected but at lower rates. PTEN-heterozygous mice develop tumors in various organs showing PTEN haploinsufficiency; while the loss of the wild-type allele was frequently observed in mouse lymphomas [16], this allele is retained in other tumor types such as thyroid or colon [17]. Furthermore, in a mouse model of prostate cancer, haploinsufficiency of the *Pten* gene leads to increased rates of tumor progression, without evidence of mutations in the retained *Pten* allele; this is similar to the situation in many human prostate cancers [18]. Retention of a WT allele is essential for the success of novel cancer therapies directed to restore PTEN activity in tumors, for example, targeting PTEN post-translational modifications as discussed below. 

Somatic PTEN mutations occur with a wide range of frequencies in sporadic tumors, with the highest frequencies observed in endometrial carcinomas and multiform glioblastoma. PTEN genetic alterations have also been reported in lymphoid malignancies, mainly in diffuse large B-cell lymphoma and T-cell acute lymphoblastic leukemia [19]. In pediatric cancer, PTEN deficiency is a common defect in juvenile myelomonocytic leukemia (JMML), with more than 65% of patients presenting a decrease in PTEN protein levels that correlates with PTEN promoter hypermethylation [20]. JMML disease often involve GM-CSF hypersensitivity and hyperactivation of the Ras pathway; while driver mutations in Ras or Nfl1 are responsible of the initialization of this process, the timing of PTEN loss might determine the disease severity [21]. 

In addition to genetic alterations, PTEN protein levels can be regulated by the epigenetic silencing of PTEN, post-transcriptional regulation by non-coding RNAs, and post-translational modifications. The use of PTEN hypomorphic mouse models has revealed that subtle variations in PTEN expression can have a dramatic effect on cancer progression [22].

Apart from PTEN being regulated at the genetic and epigenetic levels, a number of post-translational modifications are effective in inducing rapid and transient alterations in PTEN architecture, localization, and activity. These modifications include acetylation, oxidation, methylation, ribosylation, nitrosylation, phosphorylation, sumoylation, and ubiquitination (Table 1). Here, we revisit the consequences for PTEN phosphatase activity of exhibiting each of these modifications with a special focus on phosphorylation, sumoylation, and ubiquitination.

## 2. PTEN Ribosylation, Nitrosylation, and Methylation

Perhaps the least studied PTEN PTM is ADP-ribosylation (the addition of one or more ADP-ribose moieties to a protein). PTEN is a substrate for tankyrases, members of the poly(ADP-ribose) polymerase (PARP) family. Once ribosylated, PTEN is ubiquitinated by RNF146, a PAR-binding E3 ubiquitin ligase, and degraded by the proteasome. Accordingly, the depletion of tankyrases TNK1 and TNK2 in HCT116 cells results in PTEN stabilization and a reduced tumor xenograft growth [23]. 

PTEN can also be regulated in response to oxidative stress, both S-nitrosylation—a covalent modification of cysteine residues by nitric oxide (NO)—and oxidation—mediated by reactive oxygen species (ROS)—affect PTEN enzymatic activity and stability. The high abundance of ROS in cancer cells leads to PTEN oxidation: a covalent disulfide bond is formed between the critical cysteine 124 in the catalytic pocket and the neighboring C71 which results in PTEN inactivation [24,25]. As for S-nitrosylation, low NO levels promote the selective S-nitrosylation of PTEN at C83, inhibiting PTEN activity and therefore triggering Akt signaling [26]. Nitric oxide can also impact on PTEN stability as neuron treatment with NO donors enhanced PTEN ubiquitination and proteasomal degradation; it is not clear whether this destabilization is a direct consequence of PTEN nitrosylation or if it is caused by other cellular signals triggered by NO [27].

PTEN methylation has only recently been reported; an in vitro methyl transferase assay revealed that the lysine methyltransferase SYMD2 methylated PTEN K313. This methylation reduces PTEN activity [28]. R159 is methylated by protein arginine methyltransferase 6 (PRMT6), this methylation is essential for the lipid phosphatase activity of PTEN. Supporting the key role of this modification in PTEN tumor suppressor activity, methylation-deficient R159K mutations have been found in several tumor types [29]. Finally, methylation also regulates PTEN nuclear functions after genotoxic stress; NSD2 (nuclear SET domain-containing protein)-mediated methylation of K349 is involved in PTEN recruitment to DNA damage sites and is required for efficient repair of DNA double-strand breaks. This methylation therefore regulates cells’ sensitivity to DNA-damaging agents, including chemotherapeutics and radiotherapeutics [30].

## 3. C-Terminal Phosphorylation Closes PTEN

Phosphorylation is an important mechanism to regulate PTEN phosphatase activity. PTEN has a cluster of phosphorylation acceptor sites in the CT-tail that are substrates for different kinases, e.g., RhoA-associated kinase (ROCK) [31], glycogen synthase 3β (GSK3β), and casein kinase 2 [32,33]. These phosphorylations affect PTEN in different ways. 

During directed cell migration, both Rho family GTPases and PIP_3_ play crucial roles. Whereas Rho GTPases regulate F-actin polymerization and actin–myosin contractility, PIP_3_ localization at the leading edge is crucial to concentrate F-actin-rich lamellipodia and filopodia in this area [34]. In addition to controlling cell rear retraction during cell migration, RhoA regulates PTEN physical localization at the cell rear, thus restricting PIP_3_ accumulation to the leading edge. This mechanism involves the association of ROCK with PTEN, and ROCK-induced PTEN phosphorylation in four sites located in the C2 domain (S229, T232, T319, and T321; see Figure 1); these phosphorylations induce PTEN activation and translocation to the plasma membrane [31].

Apart from the C2 domain residues, the CT-tail of PTEN is phosphorylated at Ser370 and in a serine/threonine cluster (S380, T382, T383, and S385) (Figure 1). Both GSK3β and casein kinase 2 have been shown to modify these residues [32,33]. The phosphorylation of the serine/threonine cluster is required to maintain PTEN stability, as the mutation of these residues to alanine reduced PTEN half-life [35]. The phosphorylation of the CT-tail also contributes to the modulation of PTEN function, as it reduces PTEN phosphatase activity [36]. When the CT-tail is phosphorylated, PTEN adopts a closed conformation involving intra-molecular interactions that prevent association with other proteins and the plasma membrane [36,37]. On the contrary, when dephosphorylated, PTEN adopts an open/active conformation [36,37]. Although here discussed as a globally phosphorylated or dephosphorylated CT-tail, partial reductions in the phosphorylation of individual Ser or Thr residues in the CT-tail may also impair PTEN enzymatic activity [38].

The very C-terminus (residues 400 to 403) of PTEN mediates association with several proteins containing PDZ domains, such as MAGI-2 and MAGI-3 that enhance PTEN activity and localization to plasma membrane [39]. The CT-tail phosphorylation-less T382A/T383A mutant was unstable but exhibited enhanced PTEN binding with MAGI-2 and with the cell membrane [39]. Taken together, these data suggest that C-terminal phosphorylation regulates PTEN’s open/closed conformations, its binding to MAGI proteins, and its capacity to be activated at the cell membrane.

Other Ser/Thr PTEN phosphorylations do not affect its phosphatase activity, e.g., ATM-induced PTEN phosphorylation at Ser113 (in response to DNA damage) that promotes PTEN translocation to the nucleus and induces cell autophagy [40], or ATM phosphorylation at S398 which reduces the nuclear localization of PTEN (when previously sumoylated on K254, see below) [41]. ATM PTMs are not included in Figure 1.

PTEN can also be phosphorylated at Tyr residues, e.g., phosphorylation of Tyr240 and Tyr315 by Src reduces PTEN interaction with the plasma membrane and its activity, resulting in increased PI3-kinase/AKT activation [42,43] (Figure 1). FGFR2 can also phosphorylate PTEN at Y240, this phosphorylation is frequent in glioma patients with high PTEN and FGFR2 expressions. The irradiation of glioma cells increases pY240-PTEN nuclear localization and promotes chromatin decondensation. Enhanced chromatin accessibility facilitates homologous recombination DNA repair. This mechanism is independent of PTEN phosphatase activity and could be responsible for the therapeutic resistance to radiotherapy observed in a high proportion of glioma patients [44,45].

Src-mediated PTEN phosphorylation at Tyr155 also affects PTEN susceptibility to ubiquitination by WWP2 [46]. Finally, the Src kinase Rak phosphorylates PTEN at Y336; this phosphorylation increases PTEN stability [47]. The stability of PTEN is regulated by E3 ligase NEDD4.1-induced polyubiquitination, followed by proteasomal degradation [48]. Without Rak, the complex NEDD4.1–PTEN increases, which in turn increases PTEN polyubiquitination/degradation. Rak-phosphorylation of Y336-PTEN reduces the NEDD4.1–PTEN complex, promoting PTEN stability. Phosphorylation of Tyr336 can also be mediated by PTK2/FAK [49] (Figure 1).

## 4. Acetylation Might Activate or Inactivate PTEN

**Figure 2:**
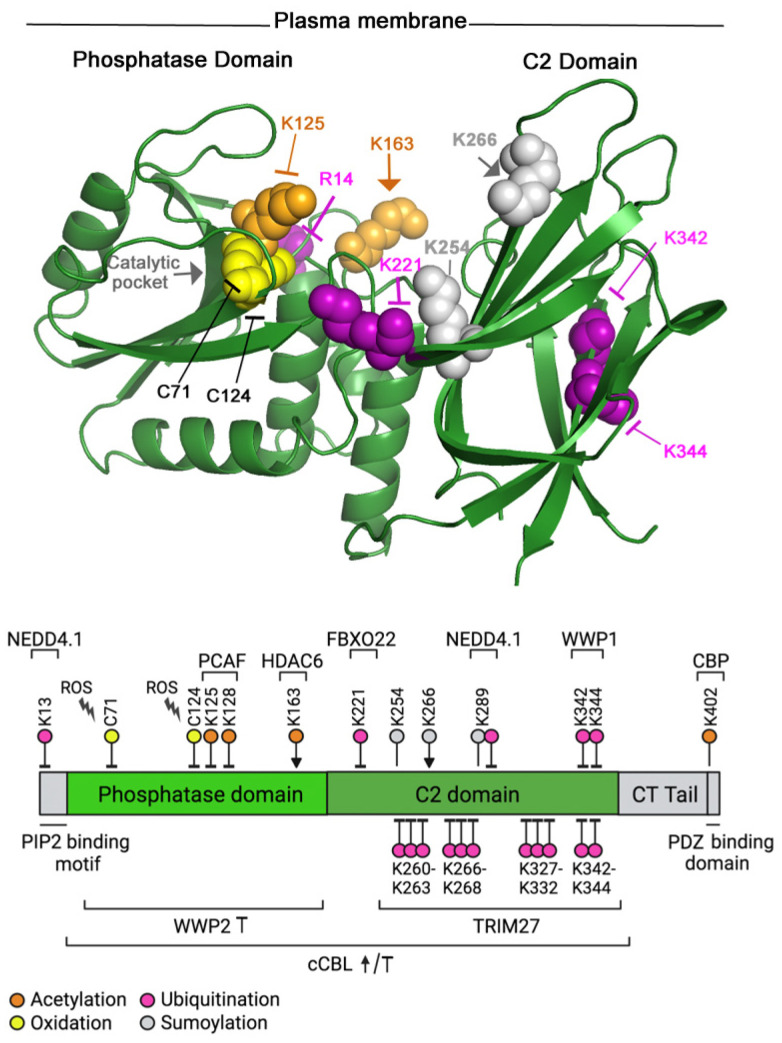
Lys163 acetylation and Lys266 sumoylation activate PTEN, but most PTEN modifications inhibit its phosphatase activity. PTEN’s structure (**top**) and the diagram of PTEN domains (**bottom**) are as in Figure 1. Post-translational modifications (PTMs) are indicated in different colors (codes along the **bottom**). PTMs activate (indicated with an arrow) or inhibit (arrows ended with a line) PTEN activity. The effect of some PTMs on PTEN is unknown (arrows without end). The enzymes regulating PTEN PTMs are indicated in the diagram. Most PTMs shown are inhibitory, as only Lys163 acetylation and Lys266 sumoylation increase PTEN action. The N-terminal PIP_2_-binding motif, as well as the C-terminal (CT)-tail and PDZ binding domains, are shown only in the diagram (in grey), as they are not resolved in the structure, i.e., neither K13 nor K402 PTMs are shown in the structure; R14 is indicated on the structure to offer an idea of where K13 localizes. The region including K289 is also unresolved and not shown on the structure. The residues modified by WWP2 or cCBL are presently unknown, these E3 ligases and the multiple residues modified by TRIM27 are shown in the bottom part of the diagram.

Another well-established post-translational modification is lysine acetylation. Acetylation was first described for histones, but in recent years more than 100 non-histone proteins have been shown to be acetylated [50]. Protein acetylation is regulated by lysine acetyltransferases and deacetylases. PTEN associates with the acetyltransferase PCAF (p300/CBP-associated factor) in a growth-factor-dependent manner [51]. This association results in the acetylation of Lys125 and 128 in the PTEN catalytic domain. The acetylation of Lys125 and 128 in the catalytic pocket vicinity inactivates PTEN [51] (Figure 2). In contrast, the acetylation of Lys163 in the final region of the phosphatase domain enhances PTEN translocation to the plasma membrane and its phosphatase activity, as it impairs the intramolecular interactions that keep PTEN in a closed conformation [52]. Accordingly, the inhibition of HDAC6 histone deacetylase increased K163 acetylation, PTEN translocation to the membrane, and PTEN activity. K163 increased acetylation by treatment with HDAC inhibitors, reduced active pAKT levels, and impaired tumor growth in WT-PTEN-expressing cells compared to (acetylation-resistant) PTEN-K163R-expressing cells [52].

PTEN is also acetylated at Lys402 by CBP; Lys402 is localized in the PDZ domain-binding motif at the very C-terminal end of the protein [53] (Figure 2). This motif is involved in the interaction of PTEN with MAGI-2, MAGI-3, and hDLG [54,55], which as mentioned above modulates PTEN binding to the membrane. Mutation analysis has demonstrated that the acetylation of PTEN Lys402 increased the interaction with the PDZ domains of hDLG and MAGI-2, without significantly affecting PTEN phosphatase activity or subcellular localization [53]. Taken together, of the four residues modified, the enhanced acetylation of Lys163 by the inhibition of deacetylase HDAC6 seems to be a good approach to increase PTEN activity.

## 5. Sumoylation Brings PTEN to the Membrane

SUMO (small ubiquitin-related modifier) is a 10 kDa polypeptide that is reversibly attached to lysine residues, modifying protein characteristics such as activity, stability, and localization. Sumoylation requires an E1-activating enzyme, an E2-conjugating enzyme, and an E3 SUMO ligase (although sumoylation can be achieved without an E3) [56,57,58]. In humans, there are four different SUMO proteins; the SUMO2 and 3 proteins are highly related and, in some cases, are conjugated to different protein targets than SUMO1. The role of SUMO4 has not yet been clearly established [57]. Whereas SUMO1 only binds to its substrates once, SUMO2 and 3 form large sumoylated branches in their target molecules [57]. A single E1-activating enzyme (SAE1/SAE2 heterodimer), a single E2-conjugating enzyme (Ubc9), and several E3 ligases mediate SUMO conjugation to Lys residues [57]. Apart from the covalent binding of a SUMO polypeptide to a protein substrate, protein sumoylation is regulated by desumoylases—cysteine proteases that are also referred as SUMO-specific proteases—of which the mammalian genome has six different variants [59].

In the case of PTEN, sumoylation assays in vitro show that this protein can be modified by SUMO1 and SUMO2/3 polypeptides at several (predicted) lysine residues. The mutation of K254, K266, or K289 (located in the C2 domain of PTEN) (Figure 2) to alanine resulted in a reduction in PTEN sumoylation, suggesting that these residues are indeed SUMO acceptors [60,61]. Sumoylation might facilitate PTEN association with the plasma membrane, as demonstrated in the case of SUMO1 modification of K266 [61]. Since this lysine is located within the C2 domain, it is possible that SUMO binding blocks the intramolecular interactions of the C2 domain with the C-terminal region of PTEN, favoring the PTEN open conformation and its association with the plasma membrane [60,61]. Additionally, molecular dynamics simulations indicate that the SUMO1 modification of PTEN presents an electropositive surface that could facilitate its interaction with the negatively charged membrane phospholipids [61]. Although, K254, K266, and K289 can be linked to SUMO1, only the modification of K266 is crucial for PTEN association with the plasma membrane and correlates with increased PTEN activity [61]. It has been shown that PTEN binding to PIP_2_ in the membrane triggers an allosteric activation of the phosphatase [62]; PTEN allosteric activation could account for the increase in the activity of K266-sumoylated PTEN. In contrast, sumoylation of PTEN K254 is critical for PTEN’s action in maintaining genomic stability in the nucleus [41]. 

Although it is clear that sumoylation is key for the regulation of PTEN localization and activity, more work is needed to determine the enzymes involved in this modification, and whether the conjugation of SUMO1 or SUMO2/3 have different impacts on PTEN function. Nonetheless, the enhanced sumoylation of K266 by the inhibition of desumoylases or interference with particular E3 ligases which increase K266 sumoylation could help to rescue PTEN phosphatase activity.

## 6. PTEN Ubiquitination

Ubiquitination is a key post-translational modification regulating PTEN action. Ubiquitination might regulate its catalytic activity, degradation, and subcellular localization. Covalent binding of ubiquitin (8.6 kDa) to a Lys residue on a protein substrate requires the contribution of an E1-activating enzyme, an E2-conjugating enzyme, and an E3 ubiquitin ligase. Ubiquitination involves the formation of an isopeptide bond between the carboxyl terminus of the ubiquitin and the amino group of the Lys side chain in a substrate [63]. Linear polyubiquitins might also be formed by amide bonds formed between the C-terminal residue of ubiquitin and the N-terminal methionine of the next ubiquitin [64]. Finally, ubiquitin itself has seven lysine residues, which can act as acceptors for additional ubiquitin ligations, generating polyubiquitin branched chains [65]. Whereas Lys 48-linked polyubiquitin directs substrate proteins for proteasomal degradation (this ubiquitin linkage exhibits a higher affinity for the proteasome) [66], the majority of non-proteolytic functions of ubiquitination are associated with Lys 63-linked ubiquitin polymers [64,65,67,68].

Many E3 ubiquitin ligases have been identified in the human genome (377 different genes, thus far). E3 ubiquitin ligases can be classified into three major groups, HECT, RING, and U-box E3; each of these possesses a distinct domain composition and protein interaction region to bind to the E2 ligase [69,70]. Within RING E3 ligases, there are also two types: simple RING-finger E3s and the SKP1–Cullin–F-box (SCF) complex E3 ligases (also named Cullins) [69]. Similar to the case of protein sumoylation, ubiquitination is antagonized by deubiquitinating enzymes (DUBs, approximately 100 found in the human genome) [71]. 

In the case of HECT E3 ligases, three have been shown to ubiquitinate PTEN in vitro (WWP1, WWP2, and NEDD4.1), although with different consequences. NEDD4.1 mediates ubiquitination of PTEN Lys298 and Lys13, promoting PTEN translocation to the nucleus, which correlates with PTEN inactivation [72]. WWP2-mediated ubiquitination induces PTEN degradation [46,73]. Studies on WWP1′s mechanism of action on PTEN suggest that WWP1-mediated non-canonical K27-linked ubiquitination at Lys342 and Lys344 interferes with PTEN dimerization and localization to plasma membrane [74]. PTEN dimerization is important for the regulation of its enzymatic activity [75]. WWP1 expression levels are driven by MYC, and WWP1 regulation of PTEN is therefore particularly important in tumors harboring MYC amplification [74]. In the case of WWP1-mediated PTEN inactivation, the inhibition of WWP1 action by indole-3-carbinol, or by any other means, has been proposed as a mechanism for PTEN reactivation for MYC-driven tumors [74].

There are other PTEN ubiquitin E3 ligases such as TRIM27 (RFP), a member of the tripartite motif (TRIM) E3 family, containing a conserved motif collectively called RBCC, which includes a RING finger (R), a B-box zinc finger (B), and a coiled-coil (CC) domain. TRIM27 induces a non-canonical K27-linked ubiquitination of PTEN at multiple residues (Figure 2). These PTMs do not affect PTEN stability or cellular localization but reduce its activity, promoting an increase in AKT phosphorylation and a reduction in apoptosis [76]. F-box-only protein 22 (FBXO22), which is part of the SCF (SKP1–Cullin1–F-box) family of RING-E3 ligases, catalyzes the ubiquitination of nuclear PTEN at Lys221, inducing nuclear PTEN degradation. The role of FBXO22 as a tumor driver is supported by the frequent overexpression of this protein in cancer, and by xenograft models, where FBXO22 expression promotes tumorigenesis by degradation of nuclear PTEN [77]. 

cCBL is a RING E3 ligase that modifies EGFR (epidermal growth factor receptor) and promotes its internalization; cCBL binds to PI3-kinase regulatory subunits [78,79,80]. The incubation of normal cells with growth factors (e.g., serum or EGF) shows that a fraction of PTEN is constitutively associated to EGFR, but EGF addition induces a transient PI3-kinase and cCBL translocation to EGFR [78]. In parallel, the activation of normal cells induces complementary oscillations of PI3-kinase/AKT and PTEN activities shortly after cell activation [78]. An analysis of whether PTEN activity oscillations could be linked to PTMs indicated that PTEN inactivation coincides with an increase in its ubiquitination, while PTEN reactivation concurs with an increase in sumoylation [78]. Both processes were abrogated by cCBL depletion, which flattened both PTEN and AKT activity fluctuations [78].

Other observations support that PTEN ubiquitination and sumoylation could be linked; PTEN Lys266 and Lys289 can be both ubiquitinated and sumoylated, suggesting that ubiquitin and SUMO can compete for the same acceptor lysine [60]; a similar competition of ubiquitination and sumoylation was also observed in IκB [81]. This crosstalk between sumoylation/ubiquitination in PTEN was reinforced by the observation that PIASxα is a SUMO E3 ligase for PTEN. PIASxα-dependent sumoylation results in reduced PTEN ubiquitination, therefore increasing PTEN stability [82].

The variety of ubiquitin ligases that have been described to be involved in PTEN ubiquitination suggests that different mechanisms regulate this process (Figure 2). To characterize these mechanisms, it is useful to know which PTEN Lys residues are modified by each E3 ligase; this is not the case for cCBL or WWP2 (Figure 2). Several limitations make this characterization by mass spectrometry (ME) difficult. A low proportion of all the cellular PTEN is ubiquitinated, yielding a low amount of ubiquitinated peptides among all PTEN peptides. Additionally, ubiquitinated residues might deubiquitinate during sample processing, but the inclusion of deubiquitinase inhibitors affects the transient nature of AKT and PTEN oscillations [78]. Another limitation is the high number of Lys and Arg residues unevenly distributed in PTEN, generating many small tryptic peptides (i.e., the one encompassing K13) or some very long tryptic peptides (i.e., the one containing K298), that are undetectable by ME. Some authors have included mutations in PTEN to make long peptides shorter (i.e., in the K298-containing peptide [72]), while others define the modified Lys by mutating each candidate Lys and examining whether there is a reduction in the PTEN-ubiquitination level [77]. The latter could be a reasonable approach to identify WWP2 or cCBL target lysines in PTEN. Thus, from this section, it is concluded that a selective reduction in PTEN ubiquitination with particular E3-ligase inhibitors or by selective activation of DUBs might help to recover PTEN activity.

## 7. Enhancing PTEN Activating Modifications

Both cancer patients and PHTS patients exhibit frequent mutations in the PTEN gene [1,2,83]. A number of strategies aimed to reduce PI3-kinase pathway activity in PTEN-mutation-containing tumors have been conducted [84]. As PIP_3_ increases at the plasma membrane and activates the AKT/mTOR pathway, compounds inhibiting PI3-kinase, AKT, and mTOR have been tested in the clinic. Sirolimus, an mTOR inhibitor, has shown promising results for PHTS patients, with improvement in skin and gastrointestinal lesions [85]. For solid tumors, in addition to these treatments, everolimus and temsirolimus have been approved by the FDA for some tumor types, and other similarly acting compounds are in clinical trials [86]. Nonetheless, as mTORC1 inhibition impairs downstream S6K activation, it blocks the S6K-mediated PI3-kinase inactivation pathway, resulting in the reactivation of the PI3-kinase/AKT signaling pathway and limiting its clinical use. Here, we consider an alternative approach for patients in which at least one of the PTEN alleles is wild type (PHTS and cancer patients), and propose PTEN reactivation by the modulation of post-translational modifications as a mechanism to reduce cellular PIP_3_ levels.

The major challenge of this approach is to prove that interference with PTEN modifications does not affect the function of other cellular proteins; a thorough preliminary study is needed for each novel approach to show whether or not it is possible to selectively affect PTEN, without altering other essential cellular proteins/responses. 

**Figure 3:**
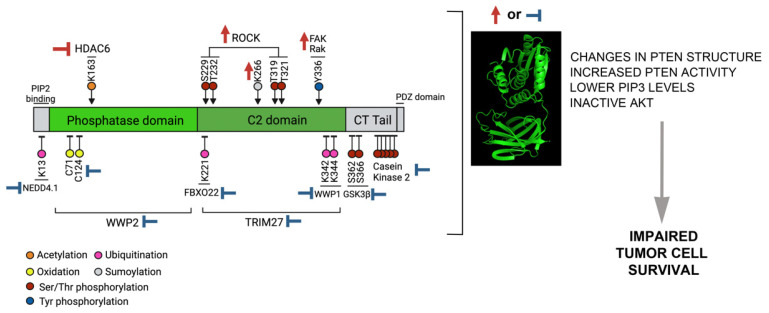
Strategies to modulate PTEN phosphatase activity. PTEN enzymatic activity in tumoral cells could be modulated by interfering with post-translation modifications. This could be achieved using strategies that either enhance PTEN-activating PTMs (upper PTMs on the left side of the figure) or inhibiting those PTMs that result in reduced PTEN phosphatase activity (lower PTMs in the PTEN scheme). Both interventions will increase PTEN’s capacity to dephosphorylate PIP_3_, therefore promoting reduced Akt activation and impaired tumor survival.

Very few modifications activate PTEN, these could be enhanced in different cancer models (with at least one WT PTEN allele) to examine whether active-AKT levels and tumor growth (in 2D or 3D) are reduced. Activating modifications include Rak and FAK-mediated Y336 phosphorylation and ROCK-induced phosphorylation of the C2 domain residues S229, T232, T319, and T321 (Figure 3). Increases in Rak, FAK, or ROCK activity, or a reduction in the phosphatases affecting these modifications, would render a global increase in these residues’ modification, in principle increasing PTEN activity. The other two modifications shown to enhance PTEN activity are Lys163 acetylation and Lys266 sumoylation. In the case of Lys163, HDAC6 inhibitors have been shown to increase Lys163 acetylation, and could be tested in different models (Figure 3). 

In the case of Lys266, more studies are needed to dissect the specificity of the sumoylation machinery in order to explain why Lys266, 254, or 289 are modified in different settings, each with different consequences. Previous studies have shown that the expression of a SUMO1–PTEN fusion protein and the depletion of SENP1 (SUMO-specific protease) increases PTEN localization at the plasma membrane and reduces both the active-AKT levels and the growth of PC3 cells [61]. The enhancement of Lys266 sumoylation might be attained by the addition of a selective SUMO-specific protease inhibitor, or by the activation of the appropriate E3 ligase, if this were to be defined.

## 8. Reduction in Inhibitory PTEN Post-Translational Modifications

Most of the PTMs examined here are inhibitory, including casein kinase 2-mediated phosphorylation of the Ser/Thr cluster (S380, T382, T383, and S385) at the PTEN CT-tail, and the GSK3β-induced phosphorylation of S362 and S366. Both modifications are required to maintain PTEN stability and the PTEN closed conformation. In this case, the recovery of PTEN activity could be achieved using selective inhibitors for these modifications. A potential strategy could be an in silico structure-based virtual screening of chemical libraries trying to find molecules that dock at these particular residues. PTEN oxidation also reduces PTEN phosphatase activity; the use of antioxidants could lead to the recovery of PTEN enzymatic activity (Figure 3).

With the exception of cCBL-mediated ubiquitination, which blocks both PTEN activation and deactivation, all the ubiquitination events revisited here act to inactivate PTEN either by promoting its degradation (WWP2, NEDD4.1, FBXO22) or by altering PTEN activity (TRIM27, WWP1). For the first group, blocking the proteasome or the different E3 ligases might be an appropriate strategy to stabilize/reactivate PTEN. In the case of TRIM27 and WWP1, they both inactivate PTEN by inducing a non-canonical K27-linked ubiquitination; if it was possible to selectively block K27-linked ubiquitination, this could help to protect PTEN activity. Alternatively, TRIM27 or WWP1 inhibitors could also be used to recover PTEN activity. The inhibition of WWP1 by indole-3-carbinol does trigger PTEN reactivation and is proposed as a therapeutic strategy for MYC-driven tumors [74]. 

TRIM27 is overexpressed in several common human tumors [87]. This ubiquitin ligase forms a complex with MAGE-L2 and ubiquitin-specific protease 7 (USP7). USP7 counteracts TRIM27 autoubiquitination, preventing its proteasomal degradation [88]. Nonetheless, USP7 targets other cellular proteins including p53, MDM2, and MDMX (negative regulators of p53) [89]. USP7 inhibitors are currently being developed by pharma companies and some have shown promising results in preclinical models in a p53-dependent manner [90,91,92]. USP7 inhibitors also mediate p53-independent effects, and these might rely on PTEN [93]. Since USP7 inhibitors destabilize TRIM27 [94], they should be effective in reducing TRIM27-induced PTEN ubiquitination and inactivation [76]. Therefore, TRIM27 destabilization by USP7 inhibitors should help in maintaining active PTEN. The potential effect of USP7 inhibitors on PTEN-dependent tumors needs to be studied.

## 9. Conclusions

Considering that PTEN loss-of-function causes PHTS and promotes cancer progression in tumors such as glioblastoma, with insufficient therapeutic armamentarium, it is necessary to delve into testing the potential of PTEN modifications as targets for PTEN reactivation. The challenge is not easy but might be worth trying given the global impact of the diseases caused by PTEN loss-of-function.

## Figures and Tables

**Table 1:** Summary of PTEN post-translational modifications showing their impact on PTEN stability or phosphatase activity and possible strategies to target these modifications.

	Residue	PTM	Biological Outcome	Possible Intervention
Enhancing activating PTEN PTMs	K163	Acetylation	Translocation to plasma membrane and increased phosphatase activity	HDAC6 inhibition
	T232, T321, S229, T319	Phosphorylation	Translocation to plasma membrane and increased phosphatase activity	ROCK activation
	K266	Sumoylation	Association to plasma membrane and increased phosphatase activity	SUMO-specific protease inhibition or activation of specific E3 ligase
	Y336	Phosphorylation	Increased PTEN protein stability	Rak or FAK activation
Inhibiting inactivating PTEN PTMs	S362, S366	Phosphorylation	Increased stability and reduced PTEN phosphatase activity	GSK3 inhibition
	S380, T382, T383, S385	Phosphorylation	Increased stability and reduced PTEN phosphatase activity	Casein Kinase 2 inhibition
	C124	Oxidation	Reduced PTEN phosphatase activity	Use of antioxidants
	K1, K289	Ubiquitination	PTEN degradation	NEDD4.1 or proteasome inhibition
	Undetermined residues in phosphatase domain	Ubiquitination	PTEN degradation	WWP2 or proteasome inhibition
	K221	Ubiquitination	PTEN degradation	FBXO22 or proteasome inhibition
	K342, K344	Ubiquitination	Reduced PTEN phosphatase activity	Indole 3 carbinol
	Multiple lysines in C2 domain	Ubiquitination	Reduced PTEN phosphatase activity	TRIM27 or USP7 inhibition
